# Palm Fruit (*Phoenix dactylifera* L.) Pollen Extract Inhibits Cancer Cell and Enzyme Activities and DNA and Protein Damage

**DOI:** 10.3390/nu15112614

**Published:** 2023-06-02

**Authors:** Hosam M. Habib, Esmail M. El-Fakharany, Hamada El-Gendi, Mohamed G. El-Ziney, Ahmed F. El-Yazbi, Wissam H. Ibrahim

**Affiliations:** 1 Research & Innovation Hub, Alamein International University (AIU), Alamein City 5060310, Egypt; hhabib@aiu.edu.eg (H.M.H.); ahmed.fawzy.aly@alexu.edu.eg (A.F.E.-Y.); 2 Protein Research Department, Genetic Engineering and Biotechnology Research Institute (GEBRI), City of Scientific Research and Technological Applications (SRTA City), New Borg El Arab, Alexandria P.O. Box 21934, Egypt; esmailelfakharany@yahoo.co.uk; 3 Bioprocess Development Department, Genetic Engineering and Biotechnology Research Institute, City of Scientific Research and Technological Applications (SRTA City), New Borg El Arab, Alexandria P.O. Box 21934, Egypt; elgendi1981@yahoo.com; 4 Dairy Science and Technology Department, Faculty of Agriculture, Alexandria University, Alexandria P.O. Box 21545, Egypt; elziney@yahoo.com; 5 Department of Pharmacology and Toxicology, Faculty of Pharmacy, Alexandria University, Alexandria 21521, Egypt; 6 Faculty of Pharmacy, Alamein International University (AIU), Alamein City 5060310, Egypt; 7 Department of Nutrition and Health, College of Medicine and Health Sciences, United Arab Emirates University, Al Ain P.O. Box 15551, United Arab Emirates

**Keywords:** palm fruit pollen, antibacterial activity, antioxidant capacity, DNA-protective, human pathogens, apoptosis

## Abstract

Palm fruit pollen extract (PFPE) is a natural source of bioactive polyphenols. The primary aim of the study was to determine the antioxidant, antimicrobial, anticancer, enzyme inhibition, bovine serum albumin (BSA), and DNA-protective properties of PFPE and identify and quantify the phenolic compounds present in PFPE. The results demonstrated that PFPE exhibited potent antioxidant activity in various radical-scavenging assays, including (2,2-diphenyl-1-picrylhydrazyl) (DPPH^•^), 2,2-azino-bis-3-ethylbenzothiazoline-6-sulfonic acid (ABTS^•^), nitric oxide (NO), ferric-reducing/antioxidant power (FRAP), and total antioxidant capacity (TAC). PFPE also displayed antimicrobial activity against several pathogenic bacteria. Similarly, PFPE reduced acetylcholinesterase, tyrosinase, and α-amylase activities. PFPE has been proven to have an anticancer effect against colon carcinoma (Caco-2), hepatoma (HepG-2), and breast carcinoma (MDA) cancer cells. Apoptosis occurred in PFPE-treated cells in a dose-dependent manner, and cell cycle arrest was observed. Furthermore, in breast cancer cells, PFPE down-regulated Bcl-2 and p21 and up-regulated p53 and Caspase-9. These results show that PFPE constitutes a potential source of polyphenols for pharmaceutical, nutraceutical, and functional food applications.

## 1. Introduction

Palm fruit pollen has been a natural product used in traditional medicine for many centuries [1]. It is a derivative of the male flower of date palm trees and is rich in several bioactive compounds, such as flavonoids, phenolic acids, and sterols [2]. Recently, there has been increasing consideration for the potential health benefits of palm fruit pollen, and numerous studies have examined its pharmacological properties [3,4,5].

Several studies have demonstrated that the anticancer activity of (PFPE) possesses anticancer activity. For instance, one study conducted by Majumder et al. (2022) [6] demonstrated that PFPE can inhibit the growth of breast cancer cells in vitro and induce apoptosis in a dose-dependent manner. This study suggested this activity may be attributed to the high content of bioactive compounds, such as polyphenols and flavonoids, in PFPE. Similarly, Kadry et al. (2019) [7] reported the anticancer activity of PFPE on liver cancer cells, showing that it inhibited their growth and proliferation and induced apoptosis in a dose-dependent manner. Other studies [8,9,10,11] have also demonstrated the potential of PFPE as an anticancer agent. However, further research is needed to fully understand the mechanisms of action of PFPE’s anticancer activity and to evaluate its safety and efficacy in human clinical trials. Overall, these findings suggest that PFPE may hold promise as a natural product for the prevention and treatment of various types of cancer.

Furthermore, PFPE exhibits several health benefits, including potent antioxidant properties that can protect cells from oxidative stress and prevent cellular damage [12,13]. Studies have shown that PFPE can scavenge free radicals and inhibit lipid peroxidation, which is a key mechanism underlying many progressive diseases [4,14].

PFPE also possesses antidiabetic properties, which can improve insulin sensitivity and control blood sugar levels [15,16]. Animal models of diabetes have demonstrated that PFPE can diminish blood glucose and enhance glucose tolerance [17]. Furthermore, PFPE exhibits anti-inflammatory activity, which is a key mechanism for many chronic diseases. Several studies have verified that PFPE can impede the formation of cytokines and reduce the expression of inflammatory markers, suggesting its potential as a natural anti-inflammatory agent [18,19,20]. In addition, PFPE has immunomodulatory properties that can help normalize the immune system and improve immune function [6,15]. Studies have shown that PFPE can modulate the production of cytokines and chemokines, which are key regulators of immune response [21].

PFPE is a natural product with a broad range of health benefits. While more research is required to fully understand its pharmacological properties and mechanisms of action, the existing evidence suggests that it may have the potential to be an antioxidant, anti-inflammatory, immunomodulatory, antidiabetic, and anticancer agent [3,22,23,24,25,26,27,28,29].

Nevertheless, most of the bioactivities of PFPE were revealed in this study for the first time. Consequently, the principal objective of this investigation was to explore the functional health effects of PFPE on protein and DNA damage, in addition to the activities of the enzymes α-Amylase, tyrosinase, and acetylcholinesterase, and antimicrobial activities that have been connected to various diseases. Additionally, the effects of PFPE on the propagation and apoptosis of hepatic, colorectal, and breast cancer cells were considered. 

## 2. Materials and Methods

### 2.1. Study Materials

The study used Khalas variety palm fruit pollen contributed by the Al Foah Company, Al Ain, UAE. Other materials used in the study include vitamin C (VC), TPTZ, rutin, sodium nitroprusside, sodium acetate buffer, iron (III) chloride, FeSO_4_, DPPH^•^, 4-amino benzene sulfonic acid, glacial acetic acid, ACS reagent, H_2_SO_4_, NaH_2_PO_4_, (NH_4_)2MoO_4_, H_2_O_2_, polysaccharide agarose, MTT, trypsin/ethylenediaminetetraacetic acid, DMSO, protease-free (RNase A DNase), (EB/AO), (PFA), and pBR322 plasmid DNA bought from Millipore Sigma Chemical Co. (Louis, MO, USA). Dulbecco’s Modified Eagle Medium (DMEM) and Gibco RPMI-1640 were bought from Thermo Fisher Scientific (Waltham, MA, USA). Normal somatic cells (HSF), breast carcinoma (MDA), colon carcinoma (Caco-2), and hepatoma (HepG-2), cells were obtained from (ATTCC), the American Type Culture Collection (Manassas, VA, USA).

### 2.2. Methods

#### 2.2.1. Plant Material Extraction and Preparation

Phenolic compounds were extracted for HPLC analysis using accelerated solvent extraction (ASE) with Dionex Co.’s Thermo Scientific ASE 350 (168 Third Avenue, Waltham, MA, USA). Stainless steel extraction cells (11 mL) and amber collection vials (40 mL) were utilized, along with ASE Prep DE. The extraction was conducted at 25 °C with a pressure of 1500 psi, a static time of 5 min, 4 static cycles, a 75% flush, and a 90 s purge. The entire phenolic fraction was eluted with 300 mL of methanol, then dried under reduced pressure at 50 °C. The resulting residue was suspended in 5 mL of distilled water and extracted with 5 mL × 3 of diethyl ether. The ether extracts were combined, concentrated under nitrogen, and dissolved in 1 mL of a 50:50 (*v*/*v*) methanol:water solution. Finally, all extracts were filtered using a 0.45 µm mesh and subjected to HPLC analysis.

#### 2.2.2. Total Phenolic Content (TPC) Assay

The TPC of the methanol:water PFPE solution was determined by applying the spectrophotometric analysis through Folin–Ciocalteu’s phenol reagent, as described previously [30]. Using a standard curve for TPC, a gallic acid standard solution with concentrations ranging from 0 to 100 mg/L was utilized. The total phenolics in the PFPE were reported as mg of gallic acid equivalent (GAE) per 100 g of PFPE.

#### 2.2.3. Total Flavonoids Content (TFC) Assay

The TFC of the PFPE was evaluated utilizing the colorimetric technique explained earlier [31]. An aliquot of PFPE or standard solution was mixed with water and a 5% NaNO_2_ solution. After 6 min, 10% AlCl_3_ solution was added, followed by the addition of 1 M NaOH solution after 5 min. The total volume was then made up to 2.5 mL with water, and the absorbance was measured at 510 nm against a blank. The TFC was reported in terms of mg rutin equivalent (RE) per 100 g of PFPE. Rutin was used as the reference standard as it is a commonly found flavonoid in food sources.

### 2.3. Antioxidants Activity 

#### 2.3.1. DPPH^•^- Free Radical-Scavenging Evaluate

A DPPH^•^- assay was performed by testing the activity of vitamin C (VC), PFPE, and rutin at varying concentrations ranging from 0.1 mg/mL to 5 mg/mL against 1,1-diphenyl-2, picrylhydrazyl radicals [32]. The percentage inhibition of these radical compounds was determined using Equation (1):

% Inhibition DPPH^•^ = (Abs. control − Abs. sample)/(Abs. control) × 100 (1)

The absorbance of the DPPH^•^ mixture without the test sample was considered as the Abs. control test. Absorbance was measured with a Varian CARY 50 Scan UV–VIS Spectrophotometer equipped with a Cary 50 Microplate Reader (Varian, Inc., Walnut Creek, CA, USA).

#### 2.3.2. ABTS^•^- Free Radical-Scavenging Quantitative Analysis

The ABTS^•^- free radical-scavenging assay was conducted to evaluate the free radical-scavenging capacity of VC, PFPE, and rutin at different concentrations (0.1 mg/mL to 5 mg/mL). The ABTS^•^ radicals are employed as a model system to assess the antioxidant activity of the compounds [33]. The percentage inhibition of the ABTS^•^ radicals by the test compounds was calculated using Equation 1, as described earlier.

#### 2.3.3. Ferric-Reducing/Antioxidant Power (FRAP) Analysis

The ferric-reducing/antioxidant power (FRAP) assay was carried out on VC, PFPE, and rutin at varying concentrations (0.1 mg/mL to 5 mg/mL) using a previously reported method [34]. The FRAP reagent was prepared by mixing TPTZ (10 mM mixture) in 40 mM HCl, FeCl_3_ (20 mM), and acetate buffer (pH 3.6, 0.3 M) in a ratio of 1:1:10 (*v/v/v*). Next, 1 mL of the test sample was mixed with 2 mL of freshly prepared FRAP reagent, and the solutions were incubated for 30 min at 37 °C. The absorbance of the resulting mixture was measured at 593 nm. Deionized water was employed as a blank, and FeSO_4_ was used to establish the calibration curve. The FRAP values were expressed as µmol of Fe (II).

#### 2.3.4. Nitric Oxide Radical (NO) Test

The inhibition of the NO radical on the activities of VC, PFPE, and rutin was evaluated using the Griess reaction at various concentrations ranging from 0.1 mg/mL to 5 mg/mL, following a previously reported method [35]. The reaction mixture contained 2 mL of sodium nitroprusside (10 mM), 0.5 mL of saline phosphate buffer, and 0.5 mL of the test samples. The mixtures were incubated for 150 min at 25 °C. After incubation, 0.5 mL of the reaction mixture was combined with 1 mL of sulfanilic acid reagent (0.33% in 20% glacial acetic acid). Following a 5 min wait, 1 mL of naphthyl ethylene diamine dihydrochloride (0.1%) was added. The mixtures were left for 30 min at room temperature, and the absorbance was measured at 540 nm. The percentage of nitrites was calculated using Equation 1, as described earlier.

#### 2.3.5. Total Antioxidant Capacity (TAC)

The (TAC) assay was carried out on VC, PFPE, and rutin at varying concentrations (0.1 mg/mL to 5 mg/mL) according to a previously reported method [36]. Briefly, a reagent containing sodium phosphate (28 mM), sulfuric acid (0.6 M), and ammonium molybdate (4 mM) was added to 0.1 mL of the test samples, and the mixture was incubated at 95 °C for 90 min. After cooling, the absorbance of the mixture was measured at 695 nm. A higher absorbance value indicates a greater antioxidant capacity of the tested samples.

### 2.4. Enzyme Inhibitory Activity 

#### 2.4.1. The assay for Inhibiting Tyrosinase Enzyme Activity

To assess the inhibition of tyrosinase activity, a previously reported method was followed in this study [37]. A mixture of L-tyrosine solution (4 mL) and phosphate buffer (20 mM pH 6.8) was added to VC, rutin, PFPE, or kojic acid at concentrations ranging from 0.1 mg/mL to 5 mg/mL. The mixture was incubated at 37 °C for 10 min, and then 1 mL of 50 units/mL mushroom tyrosinase liquefied in 0.2 M, pH 6.8 phosphate buffer was added. The resulting mixture was incubated for an additional 10 min, and the absorbance was measured at 475 nm. Ethanol (50%) was used as a blank, while 1 mL of deionized water was a control. The percentage of tyrosinase inhibition activity was calculated using Equation (1).

#### 2.4.2. The Assay for Inhibiting Porcine α-amylase Enzyme Activity

In this study, the inhibition activity of porcine α-amylase was evaluated following a previously reported method [38]. Sodium phosphate (0.02 M pH 6.9) was mixed with 50 µL of PFPE, VC, rutin, or acarbose (used as a positive standard) at dilutions ranging from 0.1 mg/mL to 5 mg/mL, along with sodium chloride (6 mM) and 13 units/mL α-amylase solution. The combination was incubated at 25 °C for a specific period, following which starch solution mixed with sodium phosphate (0.02 M pH 6.9) and sodium chloride (6 mM) was added and incubated at 25 °C for 10 min. After the incubation, 1 mL of dinitrosalicylic acid (color reagent) was included in the mixture at 100 °C for 10 min to stop the reaction, followed by chilling to 25 °C. The absorbance of the resulting combination was measured at 540 nm, after adding 1 mL of deionized water. The percentage of inhibition activity of porcine α-amylase was calculated using Equation (1).

#### 2.4.3. The Assay for Inhibiting Acetylcholinesterase Enzyme Activity

To perform the test, a mixture of 25 µL of 0.28 U/mL of acetylcholinesterase, 325 µL of 0.05 M Tris–HCl buffer pH 8, and 100 µL of PFPE, VC, rutin, or galantamine (used as a positive standard) at concentrations ranging from 0.1 mg/mL to 5 mg/mL was prepared. The mixture was then incubated at room temperature for 15 min. After this, 475 µL of 3 mM DTNB solution and 75 µL of acetylcholine iodide 15 mM were added to the mixture and incubated for an additional 30 min at room temperature. Finally, the absorbance of the mixture was measured at 405 nm, and the percentage of inhibition activity of acetylcholinesterase was calculated by Equation (1), as explained earlier [32].

### 2.5. Assay for DNA Damage Caused by Free Radicals

For the test, a combination of 4 µL of PFPE, rutin, or VC at dilutions ranging from 0.1 mg/mL to 5 mg/mL, along with 6 µL of H*_2_*O*_2_* 30%, 6 µL of PBS buffer, and 0.1 µg of plasmid pBR322 DNA dissolved in 1 µL of PBS pH 7.4 50 mM was prepared. The prepared samples were then exposed to UV irradiation for 5 min at 25 °C using an intensity of 25 W cm^−2^ at 312 via a transilluminator UV TFM-26 (UVP, Upland, CA, USA). Once the reaction was completed, the tested samples were electrophoresed through a polysaccharide agarose 0.8% gel, and we stained the electrophoresed gel with ethidium bromide. The electrophoresed gel was analyzed, and we captured photographs of the electrophoresed gel using Image Lab 4.1 software, version 6.1.0. (Bio-Rad, Hercules, CA, USA). This method allows us to detect any DNA damage caused by free radicals, and the use of different concentrations of VC, rutin, or PFPE allows us to determine their ability to prevent or reduce such damage. The use of electrophoresis and staining methods provides a visual representation of the extent of DNA damage and allows for quantification of the results [39].

### 2.6. Assay for Protein Oxidation Produced by AAPH

To perform the experiment, bovine serum albumin (BSA) at a concentration of 0.5 mg/mL was incubated with 20 mM AAPH in the presence or absence of VC, rutin, or PFPE at concentrations ranging from 0.1 mg/mL to 5 mg/mL. The mixture was then incubated in a shaking water bath for 30 min at 37 °C. A control sample not including AAPH was also prepared. After incubation, the mixture was loaded onto an SDS-PAGE 10% gel under reduction conditions for 5 min at 100 °C. Images of the stained gel were captured using a Chemi-Doc MV gel documentation system (Bio-Rad, Hercules, CA, USA). The intensity of the bands was determined to quantify the aggregate of protein damage for all bands employing Image Lab 4.1 Software version 6.1.0 (Bio-Rad, Hercules, CA, USA). The method allows for detecting protein oxidation induced by AAPH and determining the ability of VC, rutin, or PFPE to prevent or reduce such damage. The use of SDS-PAGE and staining methods provides a visual representation of the extent of protein damage and allows for quantification of the results [39].

### 2.7. Antimicrobial Activity

#### 2.7.1. Test for Susceptibility by Disk Diffusion

In this study, the agar-well diffusion approach was used to estimate the antimicrobial potential of PFPE against several social pathogens, including *Pseudomonas aeruginosa* (ATCC 27853)*, Streptococcus mutans* (ATCC 25175)*, Escherichia coli* (ATCC 25922)*, Staphylococcus aureus* (ATCC 25923)*, Salmonella typhimurium* (ATCC 14028)*,* and *Candida albicans* (ATCC 10231). The pre-cultures of these pathogens were prepared through separate cultivation on Muller–Hinton broth at 37 °C for 24 h. Three wells were created on the agar plates, where 100 µL of each concentration of PFPE (5, 10, and 20 mg/well) were inoculated separately into each well. After cultivation at 37 °C for 24 h, the developed inhibition zones (Halo zones) around wells indicated positive results. Amoxicillin (AX-25) and ampicillin (AM-10) were included as reference antibacterial drugs, while clotrimazole (CLT) and amphotericin-B (AmB) were applied as reference antifungal drugs [40].

#### 2.7.2. Assay of Minimum Inhibitory Concentrations (MICs)

The (MIC) for PFPE was calculated through the microdilution assay. Based on the agar-well diffusion results, 100 µL from the PFPE-susceptible pathogens was inoculated separately into a 96-well cell culture plate. For each pathogen, 100 µL of serially diluted PFPE (5 concentrations of 0.625, 1.25, 2.5, 5, and 10 mg/well) was included to an ending volume of 200 µL/well. After incubation for 24 h at 37 °C, the bacterial growth was measured through a microplate reader at 600 nm. The lowest dose in g/mL that prevented observable cell growth was expressed as the MIC. This assay aimed to verify the antimicrobial potential of PFPE against various human pathogens and to calculate the MIC for PFPE, which is the minimum concentration of the substance essential to inhibit the progression of the pathogen [41].

### 2.8. Anticancer Activity

#### 2.8.1. Cytotoxicity of PFPE on Normal Cells

In this study, normal HSF cells were planted onto a 96-well microplate and learned overnight in a DMEM-supplemented medium. The PFPE was then added to the cells at different concentrations, varying from 100 to 3200 µg/mL, and incubated for 24 and 48 h. After washing the cells to remove debris and dead cells, 0.05% MTT solution was added to each well and incubated for 3–5 h at 37 °C. The MTT liquid was then replaced with DMSO, and the absorbance was measured at 570 nm. The safe dosage (EC_100_) and half maximal inhibitory concentration (IC_50_) of the PFPE were determined by applying GraphPad Prism Version 7.0 software. The EC_100_ is the concentration of the extract that produces 100% cell survival, while the IC_50_ is the concentration that produces a 50% reduction in cell viability [42].

#### 2.8.2. Anticancer Activity of PFPE against Cancer Cells (MTT) Assay

The antitumor properties of PFPE were evaluated against three different cancer cell lines: HepG-2, MDA, and Caco-2. The cells were overlaid in 96-well plates and incubated for 24 h before treatment. While Caco-2 and MDA cells were maintained in a supplemented DMEM medium through 10% FBS, HepG-2 cells were kept in RPMI-1640 without 10% FBS. The PFPE was included in the cells at several concentrations, ranging from 100 to 3200 µg/mL. The cells were then incubated for 24 and 48 h at 37 °C and 5% CO_2_. The cytotoxicity of the PFPE on the cancer cells was strongminded using the MTT assay, and the values of EC_100_ and IC_50_ were regulated using GraphPad Prism 7.0 software. In addition to determining the cytotoxicity of the PFPE, the selectivity index (SI) was calculated. The SI is outlined as the ratio of the IC_50_ on normal (HSF) to the IC_50_ value of each cancer cell line. This indicates the specificity of the extract toward the cancer cells and its potential as a selective anticancer agent. Furthermore, the impact of PFPE on the morphology of the treated MDA cells was inspected using phase-contrast microscopy at various concentrations of 100, 200, and 400 g/mL [43].

#### 2.8.3. Nuclear Staining Analysis

To test the capacity of PFPE to induce apoptosis in HepG-2 and MDA cells using fluorescent nuclear staining techniques, the EB/AO and the PI dye were both employed to stain the cells. MDA cells were learned on a sterile 24-well plate and bottled with PFPE at 100, 200, and 400 µg/mL doses. Next, after incubating for 48 h, the cells were fixed with 4% paraformaldehyde and dyed with PI (10 µg/mL) or EB/AO (100 µg/mL to each dye) for 20 min. The stained cells were then observed and imprisoned using a fluorescent phase-contrast microscope (Olympus, Tokyo, Japan) equipped with a dichromatic mirror cut to 505 nm and an excitation filter (480/30 nm). The untreated cells were used as negative reference cells. The fluorescent nuclear staining techniques utilizing EB/AO and PI dye are standard methods for detecting apoptosis in cells. Ethidium bromide stains deadly cells with red fluorescence, while acridine orange stains live cells with green fluorescence. The PI dye is taken up by cells with damaged membranes, resulting in red fluorescence [44].

#### 2.8.4. Cell Cycle Analysis

The flow cytometry method was used to determine the effect of PFPE on the cell cycle distribution of MDA cells. The cells were handled with PFPE at doses ranging between 100, 200, and 400 g/mL for 48 h. After treatment, the MDA cells were trypsinized and resuspended in cold PBS. The cells were then fixed in 70% cooled ethanol and washed three times with cold PBS. The fixed cells were treated with PBS including 5 µg/mL RNase A at 25 °C for an hour before being stained with PI (Sigma-Aldrich, St. Louis, MO, USA) at a definitive concentration of 1 mg/mL in deionized water in the dark. The cell cycle distribution of the handled MDA cells was examined using flow cytometry (Partec, Jettingen-Scheppach, Germany) and analyzed using Cell Quist and Mod Fit version 5.1 software by reading at 488 nm after the cells were labeled with PI. The untreated MDA cells served as the control sample [45].

#### 2.8.5. Analysis of Quantitative Changes in Oncogene Expression

The PFPE involves purifying total RNAs from both untreated and treated HepG-2, Caco-2, and MDA cells using the Gene JET RNA purification kit. The extracted RNA was utilized to synthesize cDNA from mRNA, and qPCR was carried out, operating SYBR green master mix with specific primers for the genes of interest. The genes of interest include p53, Caspase-9, Bcl2, and p21, which are all contained in the control of apoptosis and cell cycle progression. The following specific primers were used for each gene: 5′-TCCGATCAGGAAGGCTA-GAGTT-3′/5′-TCGGTCTCCTAA-AAGCAGGC-3′ for p53, 5′-ATTGCACAGCACGTTCACAC-3′ 5′-TATCCCATCCCAGGAAGGCA-3’ for Caspase-9, 5′-ATGTTTTGCCAACTGGCCAAG-3′/5′-TGAGCAGCGCT-CATGGTG-3′ for Bcl2, and 5′-CCACAGCGATATCCAGACATTC-3′/5′-GAAGTCAAAGTTCCACCGTTCTC-3′ for p21. These primers were used to analyze and determine the relative changes in gene expression. The relative change in gene expression in the treated cancer cells relative to untreated cancer cells was calculated using the equation of 2−∆∆CT, which is based on the difference in threshold cycles (CTs) between the treated and untreated cells. The relative change in gene expression between treated cancer cells and untreated cells was calculated using the equation 2CT, which is based on the difference in CTs between the two groups [46].

## 3. Statistical Analysis

The research was conducted in triplicate (*n* = 3), meaning that each trial was repeated three times to ensure the reliability and precision of the results. Statistical software (SPSS for Windows, version 26, SPSS Inc., Chicago, IL, USA) was used to analyze the data. The change in mean values between sample variabilities was determined using a one-way analysis of variance (ANOVA), with a p-value of less than 0.05 considered statistically significant. Post hoc analysis was accomplished using Tukey multiple-range tests to regulate the differences between mean values. The mean values and standard deviation (SD) were intended for each parameter to report the results. The use of triplicate trials, ANOVA, and post hoc tests assists in ensuring the validity and consistency of the study’s findings [47].

## 4. Results and Discussion

### 4.1. Bioactive Compounds

The variation in the results of bioactive compounds among different studies may be attributed to several factors, such as soil type, maturity, season, fertilizer, origin, growing and storage conditions, diseases, and extraction procedures [48,49,50].

#### 4.1.1. Total Phenolics Content

The Folin–Ciocalteu reagent is a widely used method for measuring the total phenolic content of various samples, such as PFPE. The reagent reacts with phenols and other reducing substances to form blue-colored complexes that spectrophotometry can quantify. The absorbance of the complexes is proportional to the concentration of phenols in the sample, which can be expressed as gallic acid equivalents (GAEs). In this study, the Folin–Ciocalteu reagent was used to evaluate the total phenolic content of PFPE, which was found to be 1474.33 ± 11.85 mg (GAE)/100 g of PFPE, as shown in Table 1. This value is comparable to those reported by previous studies using the same method ranging from 540 to 23,774 mg (GAE)/100 g of PFPE [2,24,51,52]. The Folin–Ciocalteu reagent is a simple and reliable technique for assessing the antioxidant potential of phenolic compounds in natural products [53].

#### 4.1.2. Total Flavonoids Content

The total flavonoid content of PFPE is illustrated in Table 1. The results showed that the PFPE contained a high quantity of flavonoids, with a total flavonoid content of 771.00 ± 48.28 mg (RE)/100 g. This is consistent with previous studies that have also reported high levels of flavonoids in PFPE, which ranged from 467 to 7510 mg (RE)/100 g of PFPE [2,24,51,52]. The high flavonoid content of PFPE is particularly noteworthy given the health benefits associated with these compounds. Flavonoids have been shown to have antioxidant, anti-inflammatory, and anticancer properties, among other health-promoting effects.

#### 4.1.3. HPLC Quantification of Phenolic Acids and Flavonoids

Table 1 displays the presence of phenolic acids and flavonoids in PFPE. Phenolic acid and flavonoid standards were used to identify most of the phenolic compounds in PFPE, but some compounds that had similar chromatographic performance and phenolic peaks could not be confirmed due to the unavailability of standard compounds. The structure of phenolic compounds in PFPE was determined, and the major ingredients were found to be rutin, cinnamic acid, catechin, epicatechin, P-coumaric acid, syringic acid, gallic acid, ferulic acid, and 4-Hydroxy-3-Methoxybenzoic acid, which were 6.23 ± 0.111, 1.51 ± 0.016, 0.928 ± 0.032, 0.347 ± 0.021, 0.153 ± 0.003, 0.147 ± 0.003, 0.052 ± 0.003, 0.020 ± 0.001, and 0.020 ± 0.001 mg/100 g PFPE, respectively. The results of this investigation are consistent with previous research on the polyphenols in PFPE [2,24,54], although some differences were observed. These differences could be due to various factors, such as date fruit variety, degree of ripeness, geographical and climatic conditions, fertilization, soil, cultivation practices, and extraction method. In conclusion, the present study identified and quantified several phenolic compounds in PFPE that are known to have various health-promoting effects.

### 4.2. Antioxidants Activity

To systematically estimate antioxidant activity, it is suggested to utilize multiple methods due to the involved nature of the mechanism. In the case of assessing PFPE antioxidant activity, a range of assays was employed. Phenolic compounds are supposed to be the main contributors to this activity. Discrepancies between the findings of this study and those reported in previous literature may result from factors such as fertilizer use, crop maturity, seasonal variations, cultivation conditions, geographic location, soil composition, disease presence, storage protocols, and the methods used for extraction [32,48].

#### 4.2.1. DPPH^•^- Free Radical-Scavenging Activity

**Figure 1:**
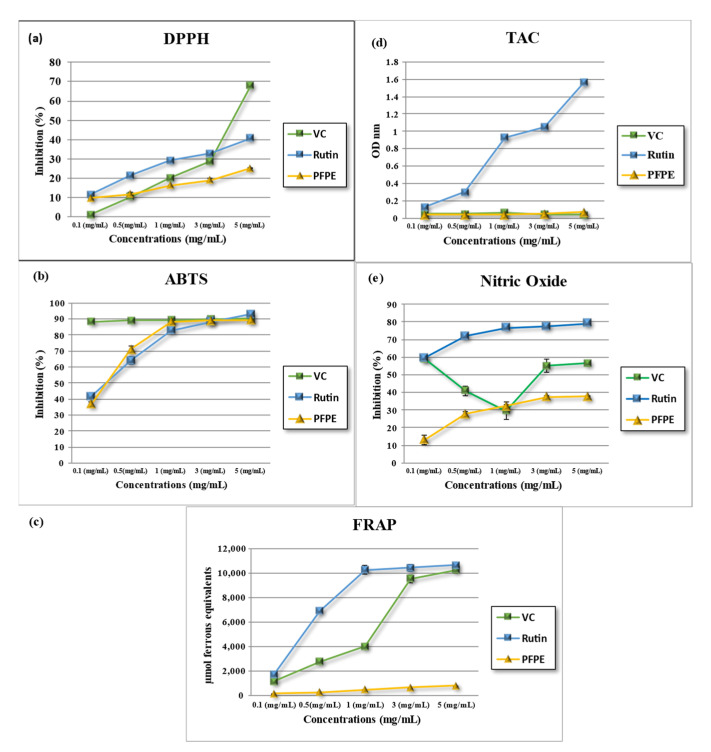
(**a**) 2,2-diphenyl-1-picrylhydrazyl, (DPPH^•^), (**b**) 2,2′-azino-bis (3-ethylbenzothiazoline-6-sulfonic acid, (ABTS^•^), (**c**) ferric-reducing/antioxidant power, (FRAP), (**d**) total antioxidant capacity (TAC), and (**e**) nitric oxide (NO) assays for (PFPE), vitamin C, and rutin at different concentrations. Data are expressed as the mean ± SD; *n* = 3.

The stable free radical DPPH^•^ is widely used in research to assess the effectiveness of natural antioxidants in vitro [32,34,38]. The results showed that the antioxidant activity of PFPE varied from 9.67 ± 0.12% to 25.18 ± 0.36%, with an IC_50_ value of 974.00 ± 28.02 µg/mL. In comparison, the IC_50_ values of vitamin C and rutin ranged from 881 ± 13.38 µg/mL to 417.80 ± 5.78 µg/mL, respectively. The results indicated that PFPE significantly inhibited the activity of DPPH^•^, as shown in Table 2 for IC_50_ and Figure 1a. The statistical analysis revealed significant inhibition (*p* < 0.05). Furthermore, the results were consistent with those reported previously in other studies [12,24,54,55]. In conclusion, the results also showed that the DPPH^•^ scavenging activity of PFPE was lower than that of vitamin C and rutin but still within a similar range. Overall, the results support the potential use of PFPE as a natural antioxidant in various applications.

#### 4.2.2. ABTS^•^- Free Radical-Scavenging Activity

This assay measures how well antioxidants can remove ABTS^•^ radicals. The samples were tested at different concentrations (ranging from 0.1 to 5 mg/mL), and the results for IC_50_ are presented in Table 2, along with Figure 1b. The results showed that PFPE’s antioxidant activity increased with concentration, reaching a maximum of 89.29 ± 0.40% at 5 mg/mL. VC and rutin also increased their antioxidant activity with concentration, reaching a maximum of 90.13 ± 0.04% and 92.69 ± 0.32%, respectively. The IC_50_ value of PFPE was 106.60 ± 1.05 μg/mL, while VC and rutin had IC_50_ values of 366.53 ± 7.46 μg/mL and 743.60 ± 30.11 μg/mL, respectively.

PFPE possesses high antioxidant activity, similar to VC and rutin. The high antioxidant activity of PFPE may be due to the phenolic compounds, which can scavenge free radicals.

#### 4.2.3. Ferric-Reducing/Antioxidant Power (FRAP) Activity

The assay measures the effect of antioxidants in reducing a ferric complex to a ferrous form. The higher the FRAP value, the higher the antioxidant activity. The FRAP values of PFPE were tested and compared with those of vitamin C (VC) and rutin, which are known antioxidants. The results are presented in Figure 1c and Table 2. The results showed that the antioxidant activity of PFPE, VC, and rutin increased concentration-dependently. The maximum FRAP value of PFPE was observed at 5 mg/mL, with a value of 788.74 ± 6.50. Similarly, VC and rutin also exhibited a concentration-dependent increase in antioxidant activity, with maximum FRAP values of 10,233.67 ± 243.72 and 10,644.00 ± 86.02, respectively. The IC_50_ value of PFPE was found to be 1671.00 ± 161.80 μg/mL, whereas the IC_50_ values of VC and rutin were 743.60 ± 30.11 μg/mL and 391.00 ± 7.46 μg/mL, respectively.

The results also show that PFPE had a lower FRAP value than VC and rutin at all concentrations tested, indicating a lower antioxidant activity. However, the FRAP value of PFPE elevated with higher concentration, suggesting a dose-dependent effect of PFPE on reducing ferric ions. The IC_50_ value of PFPE was also higher than that of VC and rutin, meaning that a higher concentration of PFPE was needed to achieve the same antioxidant effect as VC and rutin. The lower antioxidant activity of PFPE compared to VC and rutin may be due to the different chemical structures and mechanisms of action of these compounds. VC and rutin act as antioxidants that can donate electrons or hydrogen atoms to reduce ferric ions [56]. PFPE has phenolic compounds that may affect the FRAP assay differently depending on their redox potential and interaction with the ferric complex [57]. Therefore, the FRAP assay may not reflect the full antioxidant potential of PFPE.

#### 4.2.4. Total Antioxidant Capacity (TAC)

The TAC method stems from the capability of antioxidants to reduce Mo (VI) to Mo(V), which in turn produces a green phosphate/Mo(V) complex upon reaction with phosphate [58]. Results shown in Figure 1d and Table 2 exhibited that the total antioxidant capacity of PFPE increased concentration-dependently, with a maximum value of 0.067 ± 0.001 observed at 5 mg/mL. VC and rutin also exhibited a concentration-dependent increase in total antioxidant capacity, with maximum values of 0.041 ± 0.000 and 1.57 ± 0.01, respectively. The IC_50_ value of PFPE was found to be 1210.00 ± 57.32 μg/mL, whereas the IC_50_ values of VC and rutin were 2710.00 ± 13.35 μg/mL and 270.10 ± 3.51 μg/mL, respectively. These results indicate that PFPE has moderate total antioxidant capacity, which is lower than rutin but comparable to VC.

#### 4.2.5. Nitric Oxide Radical (NO)-Scavenging Activity

Nitric oxide (NO)-scavenging activity refers to the ability of a substance to neutralize or eliminate free radicals of nitric oxide, which can cause damage to cells and tissues [59]. The nitric oxide (NO)-scavenging activity of PFPE, VC, and rutin was evaluated in this study. The samples were tested at different concentrations, ranging from 0.1 to 5 mg/mL. Figure 1e and Table 2 show that the NO-scavenging activity of PFPE increased in a concentration-dependent manner, with a maximum inhibition of 37.75 ± 0.40% observed at 5 mg/mL. The NO-scavenging activity of VC and rutin also showed a similar trend, with a maximum inhibition of 56.55 ± 1.52% and 79.01 ± 0.07%, respectively. The IC_50_ value of PFPE was found to be 249.20 ± 18.67 μg/mL, whereas the IC_50_ values of VC and rutin were 2710.00 ± 13.35 μg/mL and 270.10 ± 3.51 μg/mL, respectively.

The results indicate that PFPE has moderate NO-scavenging activity, which is lower than that of VC and rutin. The observed NO-scavenging activity of PFPE may be attributed to the presence of various bioactive compounds.

### 4.3. Enzyme Inhibitory Activity 

Natural enzyme inhibitors can interact or bind to enzymes and decrease, reduce, prevent, or eliminate their activity or work normally. They can be classified into two main types: reversible inhibitors and irreversible inhibitors. They can have therapeutic applications in the treatment of several diseases [60].

#### 4.3.1. Tyrosinase Inhibition Activity

**Figure 2:**
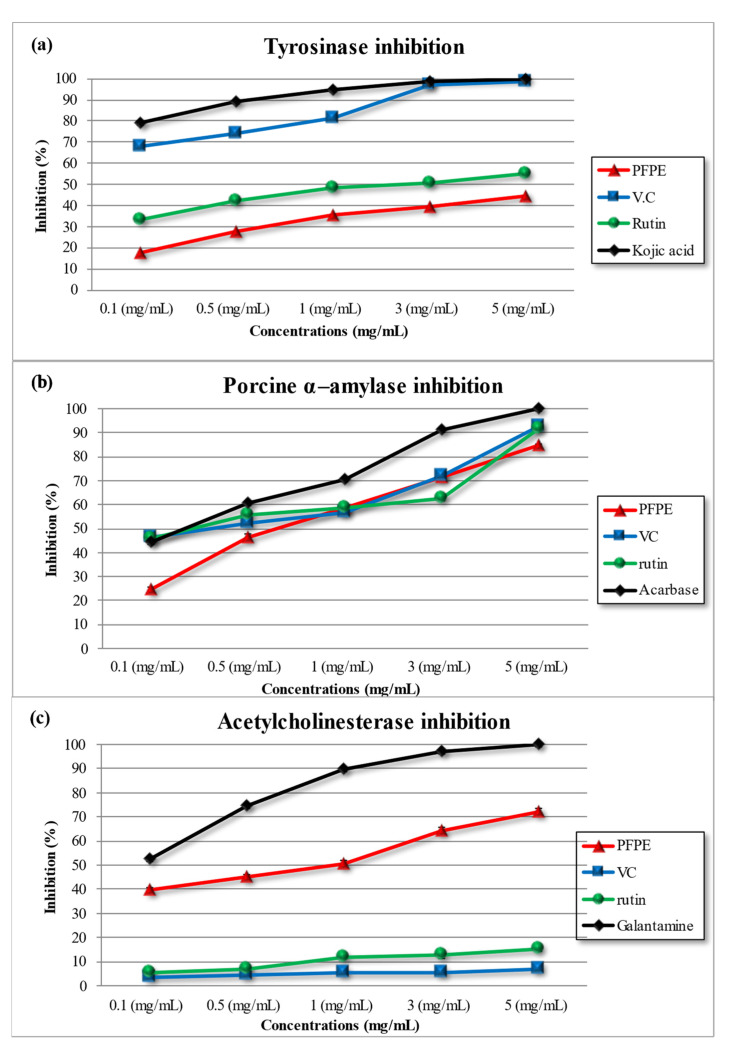
(**a**) Tyrosinase inhibitory activity, (**b**) porcine α-amylase inhibitory activity, and (**c**) acetylcholinesterase inhibitory activity for PFPE, rutin, and vitamin C at different concentrations. Data are expressed as the mean ± SD; *n* = 3.

Tyrosinase is an enzyme that contains copper and is responsible for melanin production in hair and skin, which can lead to diseases such as skin cancer and Parkinson’s disease when overproduced [32,38,61]. The study investigated the inhibitory activity of PFPE, VC, rutin, and kojic acid (a positive control) on tyrosinase, and the results in Table 3 and Figure 2a show concentration-dependent inhibitory activity for all tested compounds. At the highest concentration (5 mg/mL), PFPE inhibited 44.20 ± 0.20%, VC inhibited 98.72 ± 0.15%, rutin inhibited 55.00 ± 0.23%, and kojic acid inhibited 99.88 ± 0.11%. IC_50_ values were found to be 618.60 ± 34.54 μg/mL, 1881.00 ± 48.56 μg/mL, 494.40 ± 27.86 μg/mL, and 379.30 ± 29.28 μg/mL for PFPE, VC, rutin, and kojic acid, respectively.

The study’s results corroborate previous findings for VC, rutin, and kojic acid, while this study is the first to report PFPE’s inhibitory activity on tyrosinase [32,38,61]. The polyphenolic compounds in PFPE, such as hydroxyl groups and flavonoids, may contribute to inhibitory activity by binding to the enzyme through hydrogen bonding or chelating copper ions at the active site. Polyphenols are known to effectively inhibit the enzyme’s activity, and research has shown that PFPE polyphenols such as ferulic acid and cinnamic acid effectively inhibit and bind to several sites of the enzyme, leading to synergistic inhibition [32,62].

#### 4.3.2. Porcine α-Amylase Inhibition Activity

Amylase is responsible for breaking down starch into simpler carbohydrates. The inhibition of this enzyme may help alleviate postprandial hyperglycemia, which is beneficial for individuals [32,38,61]. The study evaluated the inhibitory activity of PFPE, VC, rutin, and acarbose on α-amylase. The results in Table 3 and Figure 2b show that all tested compounds had a concentration-dependent inhibitory effect on α-amylase. At the highest concentration tested (5 mg/mL), PFPE inhibited amylase activity by 32.26 ± 0.56%, VC inhibited it by 93.18 ± 0.18%, rutin inhibited it by 91.51 ± 0.51%, and acarbose inhibited it by 99.12 ± 0.10%. At the same time, IC_50_ was found to be 1084.00 ± 36.22 μg/mL, 97.62 ± 8.14 μg/mL, 86.76 ± 4.43 μg/mL, and 197.90 ± 17.45 μg/mL for PFPE, VC, rutin, and acarbose, respectively.

The results suggest that all tested compounds have inhibitory activity on amylase, with acarbose exhibiting the highest activity as a positive control, followed by VC, rutin, and PFPE. This is consistent with previous research that reported acarbose and VC’s inhibitory activity on α-amylase [32,38,61]. However, this study is the first to report the inhibitory activity of PFPE on amylase. The presence of polyphenolic compounds in PFPE may contribute to the inhibitory activity of α-amylase, as polyphenolic compounds are known to inhibit enzymes by binding to active sites or through non-competitive inhibition [63].

#### 4.3.3. Acetylcholinesterase Inhibition Activity

Acetylcholinesterase inhibition is an effective approach for treating neurodegenerative diseases, for example, Alzheimer’s disease (AD), due to its analgesic effects [32,38,61]. However, currently, available medications have limitations, such as side effects, rapid half-lives, bioavailability, toxicity, and gastrointestinal issues. Therefore, natural acetylcholinesterase inhibitors are being explored as a potential treatment for AD with minimal side effects [32,38,61]. The results in Table 3 and Figure 2c indicate that all the tested compounds had a concentration-dependent inhibitory effect on AChE. At the highest concentration tested (5 mg/mL), PFPE inhibited AChE activity by 17.33 ± 0.29%, VC inhibited it by 7.61 ± 0.18%, rutin inhibited it by 15.92 ± 0.13%, and galantamine (a positive control) inhibited it by 99.53 ± 0.29%. Furthermore, the IC_50_ values were found to be 653.6 ± 721.4 μg/mL, 911.7 μg/mL, and 319.6 μg/mL for PFPE, rutin, and galantamine, respectively. VC did not show significant inhibitory activity on AChE. The results indicated that all tested compounds had a concentration-dependent inhibitory effect on AChE. Galantamine was found to be the most potent AChE inhibitor among the tested compounds, consistent with previous research on its effectiveness as a therapeutic agent for AD [32,38,61]. However, this study is the first to report the inhibitory activity of PFPE on AChE, with moderate inhibition activity, suggesting its potential as a natural source of AChE inhibitors. VC did not exhibit significant inhibitory activity on AChE, possibly because of its inability to cross the blood–brain barrier [64].

### 4.4. Free Radical-Induced Damage to DNA

**Figure 3:**
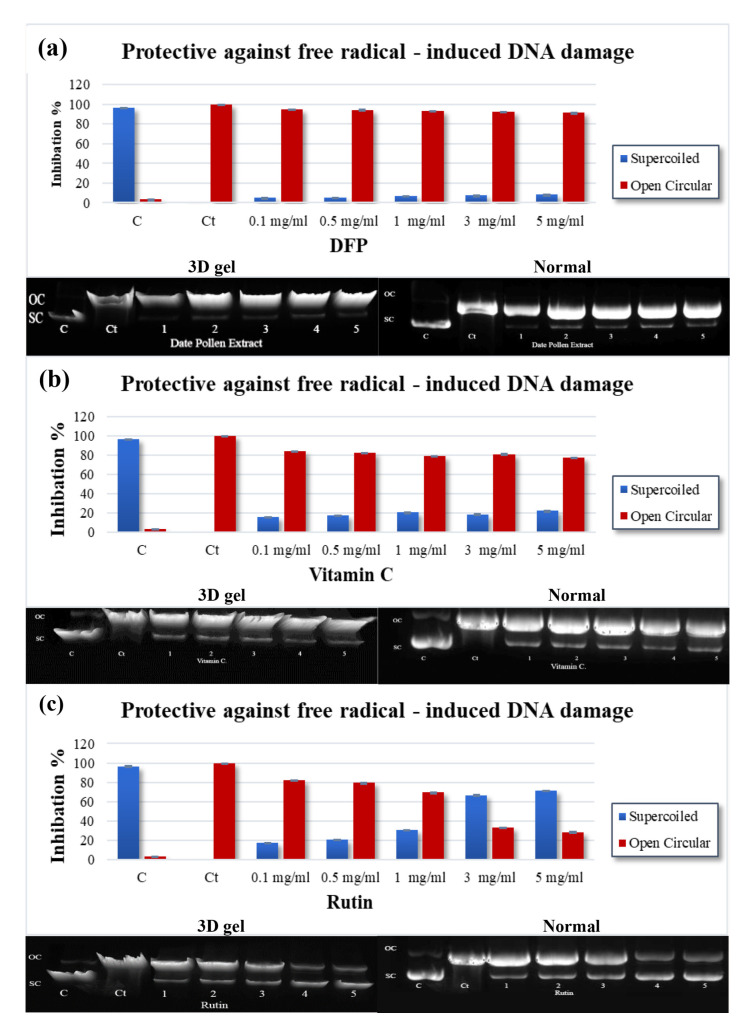
Normal gel, 3D gel, and densitometric analysis for (**a**) PFPE, (**b**) VC, and (**c**) rutin at different concentrations. C: plasmid, Ct.: plasmid + H_2_O_2_ + UV, Lanes 1–5: plasmid + PFPE, VC, or rutin at 0.1–5 mg/mL + H_2_O_2_ + UV. Data are expressed as the mean ± SD; *n* = 3.

The investigation is designed to study the ability of PFPE to protect pBR322 plasmid DNA from oxidative damage caused by UV light and H_2_O_2_. The results presented in Table 4 and Figure 3a–c show that the hydroxyl radical (OH^•^) generated by the photolysis of H_2_O_2_ caused DNA strand scission and resulted in a slow-moving open circular (OC) band and a rapid-moving native supercoiled (SC) band [32,34,38]. Pretreatment with PFPE, vitamin C, or rutin reduced DNA damage, as evidenced by an increase in the supercoiled form and a decrease in the open circular structure of DNA. The results showed that PFPE (for the first time) at concentrations ranging from 0.1 to 5 mg/mL provided a protective effect against DNA damage, with the supercoiled DNA ranging from 5.02 ± 0.05% to 8.27 ± 0.16%. The IC_50_ value for PFPE was found to be 1428.00 ± 16.57 µg/mL. Vitamin C also provided a protective effect, with the supercoiled DNA ranging from 15.90 ± 0.17% to 22.22 ± 0.13% and an IC_50_ value of 505.00 ± 21.63 µg/mL. Rutin exhibited the most protecting effect, with the supercoiled DNA ranging from 17.73 ± 0.47% to 71.49 ± 0.43% and an IC_50_ value of 15.00 ± 0.22 µg/mL.

The results suggest that PFPE, vitamin C, and rutin can protect against oxidative damage to pBR322 plasmid DNA. Rutin was found to be the most effective, followed by vitamin C and PFPE. Interestingly, the investigation also found that PFPE protected the DNA from radiation in vitro. This may be attributed to PFPE’s ability to neutralize O2^•−^ and (OH^•^) radicals. Overall, the experiment provides valuable insights into the potential antioxidant and DNA-protective properties of DSE, vitamin C, and rutin. The use of the pBR322 plasmid DNA as a model system provides a simple and effective way to assess DNA damage caused by free radicals. However, it is important to note that the results obtained using this system may not necessarily reflect the effects of these compounds on DNA damage in living cells, and further studies are needed to establish the potential health benefits of these compounds in vivo.

### 4.5. Protein Oxidation Produced by AAPH

**Figure 4:**
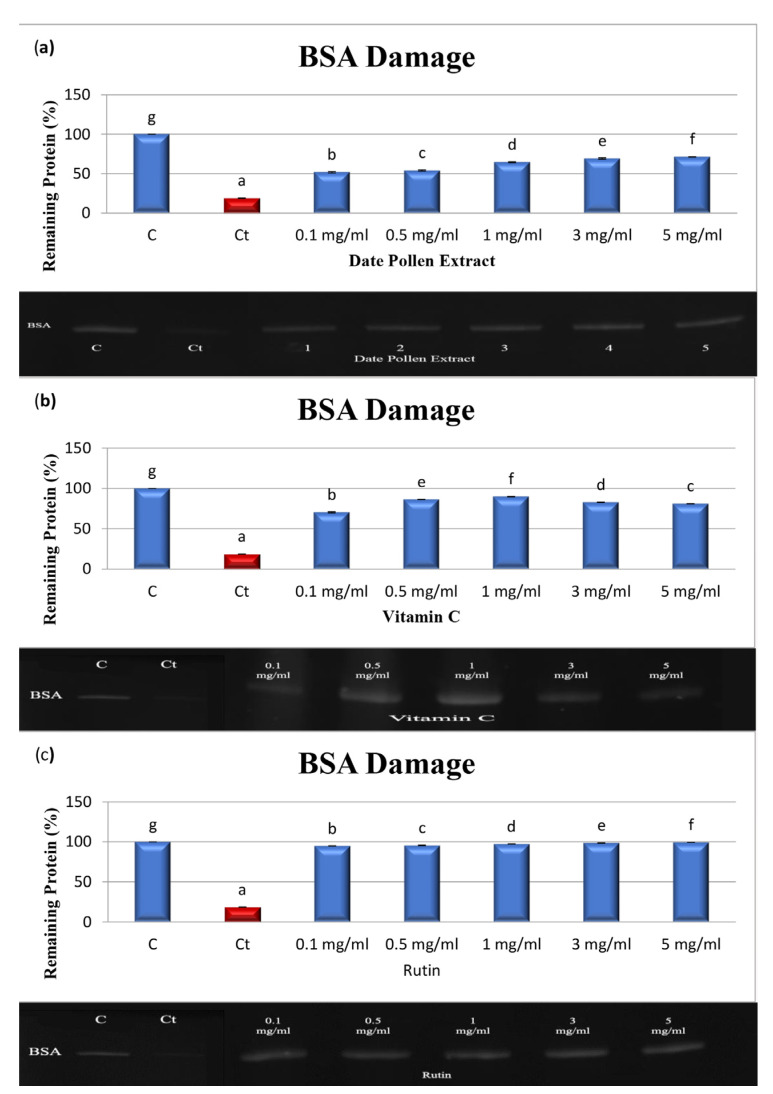
Densitometric analysis and SDS-PAGE of the effects of (**a**) PFPE, (**b**) VC, and (**c**) rutin on the oxidative damage of BSA. C: BSA, Ct: BSA + AAPH, BSA + AAPH + PFPE, VC, or rutin at concentrations of 0.1–5 mg/mL. Data are expressed as the mean ± SD; *n* = 3. Different letters in a column denote significant differences, *p* < 0.05.

Covalent modification of protein molecules results from oxidation by free radicals, leading to functional alterations of the protein. The study investigated the protective effects of PFPE, rutin, and vitamin C against protein damage caused by AAPH. The results, presented in Table 4 and Figure 4a–c, show that the band density of control BSA was 100%, while the band density of treated BSA decreased to 18.98 ± 0.32% after incubation with AAPH for 30 min [32,34,38]. Treatment with PFPE, vitamin C, or rutin at concentrations ranging from 0.1 to 5 mg/mL protected against BSA damage. The fragmentation of BSA decreased from 51.82 ± 0.94% to 71.25 ± 0.39% for PFPE, with an IC_50_ value of 64.81 ± 1.64 µg/mL. Vitamin C showed protective effects as well, with the fragmentation of BSA decreasing from 70.39 ± 0.69% to 81.08 ± 0.13% and an IC_50_ value of 25.32 ± 1.29 µg/mL. Rutin exhibited the strongest protective effect, with the fragmentation of BSA decreasing from 94.84 ± 0.26% to 99.28 ± 0.40% and an IC_50_ value of 3.68 ± 0.35 µg/mL. However, vitamin C exhibited both antioxidant and prooxidant effects at 3 mg/mL and 5 mg/mL. Notably, the study found that PFPE provided protective effects against free radical-induced protein damage for the first time. It is important to note that the study was conducted in vitro and may not fully reflect the effects of the compounds in vivo.

### 4.6. Antimicrobial Activity

**Figure 5:**
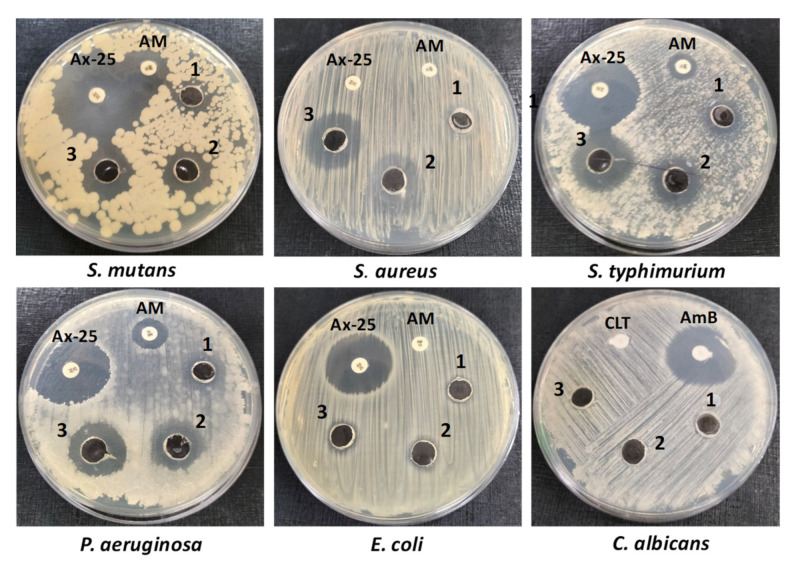
The antimicrobial activity of PFPE (1–3) at three different concentrations (5, 10, and 20 mg/well) through agar-well diffusion methods. Amoxicillin (AX-25) and amoxicillin (AM) were applied as reference antimicrobial drugs for bacteria, while clotrimazole (CLT) and amphotericin-B (AmB) were applied as antifungal references.

Antibiotic resistance is presently a significant risk to human health, and the importance of the need for influential antimicrobial drugs that have a lower possibility of inducing antibiotic resistance [65]. Natural phytochemicals are being considered due to their extensive range of biological activities and biosafety. In this investigation, the antimicrobial potential of PFPE was assessed against several pathogenic microbes [65]. The results, obtainable in Table 5 and Figure 5, exhibited that PFPE has broad-spectrum antibacterial activity against *S. aureus, S. mutans, S. typhimurium, P. aeruginosa*, and *E. coli*. However, *C. albicans* was resistant to the antimicrobial activity of PFPE at the applied concentrations. The minimum inhibitory concentration (MIC) results likewise displayed that PFPE was more effective against Gram-positive bacteria than Gram-negative bacteria, which is reliable with previous research [24]. The lowest MIC values were 12.5 mg/well for *S. mutans* and 25 mg/well for *S. aureus*, whereas the highest MIC values were 100 mg/mL against *E. coli*, followed by 50 mg/mL for both *P. aeruginosa* and *S. typhimurium*.

Gram-negative bacteria have an outer-envelope structure that selectively delays harmful agents’ access to bacterial cells, which increases their drug resistance. Hence, targeting and interfering with bacterial cell wall structure and function could be a possible antimicrobial mechanism for PFPE, as recommended by the study’s outcomes [24]. The absence of antifungal activity against *C. albicans* additionally supports the hypothesis that PFPE targets the bacterial cell wall structure and function to exercise its antimicrobial activity.

Overall, the investigation affords evidence that PFPE has broad-spectrum antibacterial activity against numerous pathogenic microorganisms. These findings suggest that PFPE has the potential to be used as a natural antimicrobial agent. 

### 4.7. Anticancer Activity

The effect of the PFPE on cell viability was examined using the MTT assay to see whether it was cytotoxic to cancer cells and safe for normal cells (Table 6). The cytotoxic effects of the PFPE were examined on the normal HSF cells, hepatoma HepG-2 cells, colon cancer Caco-2 cells, and breast cancer MDA cells to ascertain their selectivity. In contrast, the highest EC_100_ and IC_50_ values denote the greatest level of safety. Table 1 shows that the PFPE’s EC_100_ and IC_50_ values on normal cells were 3.25–4.8 and 2.86–4.98 times higher on cancerous cells, respectively, after 24 and 48 h of treatment. Table 1 further shows that the PFPE, when treated for 24 h, displayed significant anticancer activity against HepG-2, Caco-2, and MDA cells with IC_50_ values of 430.3, 394.9, and 334.5 μg/mL with SI values of 2.86, 3.12, and 3.68, respectively. After treatment for 48 h, the values of IC_50_ were determined to be 250.4, 246.9, and 223.5 μg/mL with SI values of 4.45, 4.51, and 4.98, respectively.

**Figure 6:**
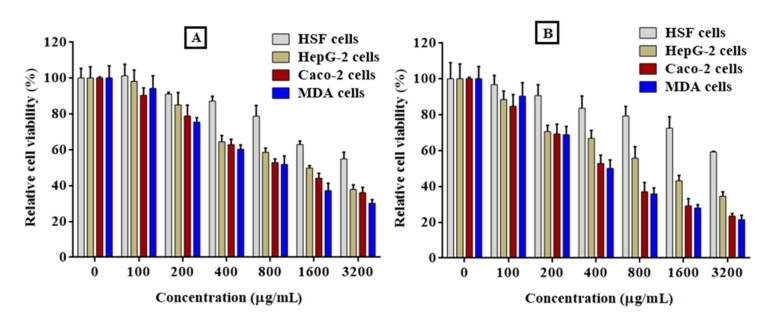
Effect of PFPE on the viability of normal and cancer cell lines. Both normal (HSF) and cancer (HepG-2, Caco-2, and MDA) cell lines were incubated with PFPE at different concentrations (0–3200 μg/mL) for 24 h (**A**) and 48 h (**B**). The test of cell viability was assayed using the MTT method. Data are expressed as the mean ± SD; *n* = 3.

**Figure 7:**
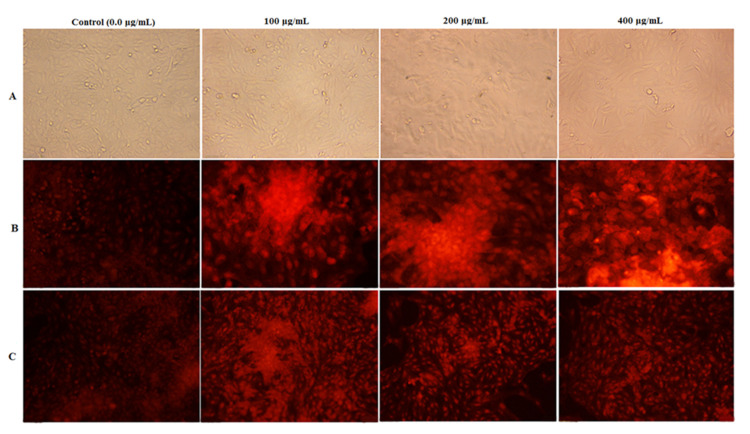
Investigation of the effect of PFPE at different ratios of 0.0 μg/mL (control), 100 μg/mL, 200 μg/mL, and 400 μg/mL on MDA cells under an inverted phase-contrast microscope. (**A**) Morphological modifications of the treated cells with PFPE. (**B**) Fluorescence images of PI staining and (**C**) fluorescence images of ethidium bromide-acridine orange staining of MDA cells.

Additionally, Figure 6 shows that the PFPE cytotoxicity was dose-dependent, with a notable improvement in the extract’s safety toward normal HSF cells and increased selectivity against cancer cells. The proportional morphological alterations and nuclear staining of MDA cells following 48 h of exposure to PFPE at 100, 200, and 400 μg/mL concentrations are depicted in Figure 7. All photomicrographs demonstrate that the therapy significantly impacted the morphology of the treated cells (Figure 7A). The morphological alterations demonstrate that PFPE causes observable cell death and modifies cell morphology dose-dependently. These alterations include blabbing, cell shrinkage, and nuclear condensation (Figure 7A). By observing the nuclear alternation in MDA cells after treatment, it was possible to provide additional evidence for PFPE’s ability to induce apoptosis, in contrast to untreated MDA cells, which exhibit barely detectable PI-positive stained cells. Figure 7B shows that the nuclei of the treated cells showed an increase in condensation and chromatin fragmentation features when the dose of PFPE was increased. Figure 7C shows that PFPE-treated MDA cells that have lost their membrane integrity have an increased incidence of apoptosis at a late stage. The treated cells exhibit orange instead of green fluorescence in negative control cells. Additionally, the number of necrotic cells rose dose-dependently, and the treated cells’ nuclei unevenly produced red fluorescence rather than green in negative reference cells. The number of necrosis cells increased, and it appeared as though some of the cells were starting to be in the decomposition process (Figure 7C).

**Figure 8:**
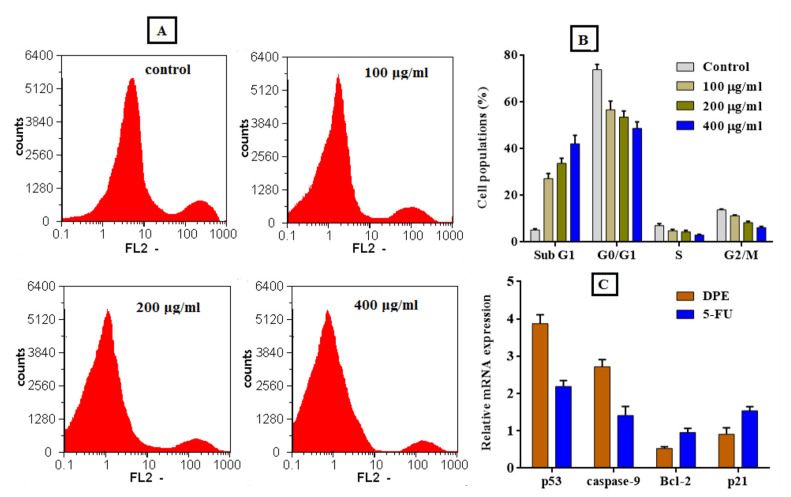
Cell cycle distribution of and gene expression profile of treated MDA cells with the PFPE at different doses for 48 h, (**A**) original flow charts of cell cycle distribution diagrams, and (**B**) quantitative distribution of PFPE-treated cells in different phases of the cell cycle in comparison with untreated (control) cells. (**C**) Relative fold change in the gene expression of P53, caspase-9, Bcl2, and p21 in the PFPE-treated cells using qPCR. Angiogenesis-related genes are evaluated in MDA cells before and after treatment with the PFPE in comparison with 5-FU for 48 h. Data are expressed as the mean ± SD; *n* = 3.

To obtain further insight into the PFPE anticancer mechanism, the arresting of the cell cycle distribution of MDA cells after treatment with the extract was examined. The cell distribution expansion in both G0/G1 and G2/M (main checkpoints phases) is enhanced by PFPE, as shown in Figure 8A,B. By increasing the treatment dose (100, 200, and 400 g/mL), the percentage of sub-G1 phase cells increased noticeably. However, as seen in Figure 8A,B, the synthesis (S) phase is reduced dose-dependently. The results show that PFPE can cause the cell cycle to be arrested in treated MDA cells as opposed to untreated cells, mostly through triggering apoptosis in a dose-dependent manner. Figure 8C also shows that the PFPE can up-regulate proapoptotic genes of p53 and Caspase-9 and down-regulate oncogenes of Bcl-2 and p21 in the treated MDA cells. The PFPE (IC_50_ dosage) is shown to have a significantly stronger activity to inhibit the expression of both Bcl-2 and p21 genes and boost p53 and Caspase-9 expression levels than more than 2-4-fold more than untreated cells and about 2-fold more than 5-FU-treated cells.

## 5. Conclusions

The current research on PFPE mainly emphasizes exploring its health benefits and some of the underlying mechanisms of action. The results from the present investigation demonstrate, for the first time, that PFPE inhibits free radical activities and DNA and protein damage, suggesting a strong potential for PFPE to protect against oxidative damage. Furthermore, DSE also inhibited acetylcholinesterase, α-amylase, and tyrosinase, whose activities have been associated with several widespread diseases. PFPE also possesses anticancer effects against MDA, HepG-2, and Caco-2 cell lines. The study also observed that PFPE could significantly up-regulate proapoptotic genes of p53 and Caspase-9 while down-regulating oncogenes of Bcl-2 and p21. Moreover, it also has activity against several human pathogens. The results suggest that PFPE could be used as an antioxidant, antimicrobial, and anticancer agent. Furthermore, PFPE could be a promising natural source for use in nutraceutical, pharmaceutical, and functional food applications.

## Figures and Tables

**Table 1:** Bioactive compounds of palm fruit pollen.

Bioactive Compounds (mg/100 g Palm Fruit Pollen)										
TPC	TFC	Gallic Acid	4-Hydroxy-3- Methoxybenzoic Acid	Catechin	Syringic Acid	Epicatechin	P-Coumaric Acid	Ferulic Acid	Rutin	Cinnamic Acid
1474.33 ± 11.85	771.00 ± 48.28	0.052 ± 0.003	0.007 ± 0.000	0.928 ± 0.032	0.147 ± 0.003	0.347 ± 0.021	0.153 ± 0.003	0.020 ± 0.001	6.23 ± 0.111	1.51 ± 0.016

All values were expressed as mean ± SD; *n* = 3. Nd: not detected; TFC: total flavonoids content; TPC: total phenolics content. The results are reported on a wet basis.

**Table 2:** In vitro antioxidant inhibition activity. IC_50_ (µg/mL) is the concentration of PFPE, VC, and rutin that can scavenge free radicals by 50%. The results are reported on a wet basis.

Radicals	Antioxidant Inhibition Activity IC_50_ (µg/mL)		
	PFPE	VC	Rutin
DPPH	974.00 ± 28.02	881.70 ± 13.38	417.80 ± 5.78
ABTS	106.60 ± 1.05	0.69 ± 0.06	6.50 ± 0.08
FRAP	1671.00 ± 161.8	743.60 ± 30.11	391.00 ± 7.46
Total Antioxidant Capacity	1210.00 ± 57.32	3005.00 ± 72.45	17,600 ± 41.64
Nitric Oxide	249.20 ± 18.67	2710.00 ±13.35	270.10 ± 3.51

All values were expressed as mean ± SD; *n* = 3.

**Table 3:** In vitro enzyme inhibition activity. IC_50_ (µg/mL) is the concentration of PFPE, VC, and rutin that can scavenge free radicals by 50%. The results are reported on a wet basis.

	Enzyme’s Inhibition Activity IC_50_ (µg/mL)					
Enzymes	PFPE	VC	Rutin	Kojic Acid	Acarbose	Galantamine
Tyrosinase	618.60 ± 34.54	1881.00 ± 48.56	494.40 ± 27.86	379.30 ± 29.28	--	--
Porcine α-amylase	1084.00 ± 36.22	97.62 ± 8.14	86.76 ± 4.43	--	197.90 ±17.45	--
Acetylcholinesterase	653.60 ± 18.54	721.40 ± 7.96	911.70 ± 17.21	--		319.60 ± 10.38

All values were expressed as mean ± SD; *n* = 3.

**Table 4:** In vitro DNA, BSA inhibition activity. IC_50_ (µg/mL) is the concentration of PFPE, VC, and rutin that can scavenge free radicals by 50%. The results are reported on a wet basis.

	DNA, BSA Damage Inhibition Activity IC_50_ (µg/mL)		
	PFPE	VC	Rutin
DNA	1428.00 ± 16.57	505.00 ± 21.63	15.00 ± 0.22
BSA	64.81 ± 1.64	25.32 ± 1.29	3.68 ± 0.35

All values are expressed as mean ± SD; *n* = 3.

**Table 5:** The antimicrobial activity of PFPE against several human pathogens expressed in inhibition-zone diameters (mm) with corresponding MIC values (mg/mL). The results are reported on a wet basis.

Organism	Control 1	Control 2	PFPE (mg/Well)			MIC (mg/mL)
			5	10	20	
*S. aureus*	−ve	−ve	12.50 ± 1.10	19.00 ± 2.10	22.00 ± 1.30	25.00
*S. mutans*	37.00 ± 2.80	18.00 ± 1.48	14.00 ± 1.50	18.00 ± 2.10	23.00 ± 1.90	12.50
*E. coli*	25.00 ± 2.40	−ve	−ve	10.00 ± 0.49	11.00 ± 0.89	100.00
*S. typhimurium*	30.00 ± 1.30	10.00 ± 0.80	13.00 ± 0.47	17.00 ± 0.47	21.60 ± 1.80	50.00
*P. aeruginosa*	28.00 ± 2.76	13.20 ± 0.68	14.20 ± 1.23	17.30 ± 2.10	20.60 ± 1.90	50.00
*C. albicans*	-	25.50 ± 2.30	−ve	−ve	−ve	-

Controls 1 and 2 are amoxicillin (25 mg/disc) and ampicillin (10 mg/disc), respectively, for bacteria, while clotrimazole and amphotericin-B at a concentration of 10 mg/disc for yeast. All values are expressed as mean ± SD; *n* = 3.

**Table 6:** EC_100_, IC_50_ (μg/mL), and SI values of the PFPE against HSF, HepG-2, Caco-2, and MDA cell lines after treatment for 24 and 48 h. The results are reported on a wet basis.

	24 h			48 h		
Cells	EC_100_	IC_50_	SI	EC_100_	IC_50_	SI
HSF	133.24 ± 4.49	1232 ± 44.93	-	123.43 ± 3.52	1114 ± 39.02	-
HepG-2	41.04 ± 1.29	430.3 ± 12.95	2.86 ± 0.11	27.05 ± 1.16	250.4 ± 11.58	4.45 ± 0.16
Caco-2	32.51 ± 1.68	394.9 ± 16.81	3.12 ± 0.12	29.36 ± 2.17	246.9 ± 25.42	4.51 ± 0.15
MDA	31.46 ± 1.74	334.5 ± 17.37	3.68 ± 0.14	25.69 ± 2.54	223.5 ± 21.72	4.98 ± 0.17

All values are expressed as mean ± SD; *n* = 3.

## Data Availability

Not applicable.

## References

[1] Waly M. (2020). Phytochemical Characterization and Health Benefits of Omani Date Pollen. FASEB J..

[2] Benouamane O., Vergara-Barberán M., Benaziza A., García-Alvarez-Coque M.C., Simó-Alfonso E., China B., Lerma-García M.J. (2022). Characterization of different cultivars of Algerian date palm (*Phoenix dactylifera* L.) leaves and pollen by comprehensive two-dimensional liquid chromatography of phenolic compounds extracted with different solvents. Microchem. J..

[3] Bentrad N., Gaceb-Terrak R., Benmalek Y., Rahmania F. (2017). Studies on chemical composition and antimicrobial activities of bioactive molecules from date palm (*Phoenix dactylifera* L.) Pollens and seeds. Afr. J. Tradit. Complement. Altern. Med..

[4] Abu-Reidah I.M., Gil-Izquierdo Á., Medina S., Ferreres F. (2017). Phenolic composition profiling of different edible parts and by-products of date palm (*Phoenix dactylifera* L.) by using HPLC-DAD-ESI/MSn. Food Res. Int..

[5] Karim K., Awad M.A., Manar A., Monia J., Karim A., Mohammed E. (2022). Effect of flowering stage and storage conditions on pollen quality of six male date palm genotypes. Saudi J. Biol. Sci..

[6] Majumder M., Nandi P., Omar A., Ugwuagbo K.C., Lala P.K. (2018). EP4 as a Therapeutic Target for Aggressive Human Breast Cancer. Int. J. Mol. Sci..

[7] Kadry M., Megeed R.M.A., Ghanem H., Abdoon A., Abdel-Hamid A.-H. (2019). Does glycogen synthase kinase-3 β signaling pathway has a significant role in date palm pollen cancer therapy?. Egypt. Pharm. J..

[8] El-Far A.H., Oyinloye B.E., Sepehrimanesh M., Allah M.A.G., Abu-Reidah I., Shaheen H.M., Razeghian-Jahromi I., Alsenosy A.E.-W.A., Noreldin A.E., Al Jaouni S.K. (2019). Date Palm (Phoenix dactylifera): Novel Findings and Future Directions for Food and Drug Discovery. Curr. Drug Discov. Technol..

[9] Abdallah W.E., AbdelMohsen M.M., Awad H.M. (2022). Phytochemical Composition, Antioxidant and Antitumor Activities of some Date Palm Pollen Extracts. Egypt. J. Chem..

[10] Teo D., Abdullah B., Zainatul N., Binti Zainol N., Hasraf N., Nayan B.M., Muhammad N.B. (2021). A Systematic Literature Review: The Effect of Date Palms (Phoenix Dactylifera) toward Breast Cancer MCF-7 Cell Line. Ann. Rom. Soc. Cell Biol..

[11] El-Far A.H., Ragab R.F., Mousa S.A. (2021). Date Palm Bioactive Compounds: Nutraceuticals, Functional Nutrients, and Pharmaceuticals. The Date Palm Genome.

[12] Karra S., Sebii H., Jardak M., Bouaziz M.A., Attia H., Blecker C., Besbes S. (2020). Male date palm flowers: Valuable nutritional food ingredients and alternative antioxidant source and antimicrobial agent. S. Afr. J. Bot..

[13] El Azim M.H.M.A. (2015). Identification Phenolic and Biological Activities of Methanolic Extract of Date Palm Pollen (Phoenix dactylifera). J. Microb. Biochem. Technol..

[14] Cazarin C.B.B., Bicas J.L., Pastore G.M., Marostica M.R. (2021). Introduction. Bioactive Food Components Activity in Mechanistic Approach.

[15] Abdel-Shaheed M.M., Abdalla E.S., Khalil A.F., El-Hadidy E.M. (2021). Effect of Egyptian Date Palm Pollen (*Phoenix Dactylifera* L.) and Its Hydroethanolic Extracts on Serum Glucose and Lipid Profiles in Induced Diabetic Rats. Food Nutr. Sci..

[16] Bentrad N., Hamida-Ferhat A. (2020). Date palm fruit (Phoenix dactylifera): Nutritional values and potential benefits on health. The Mediterranean Diet.

[17] Mia M.A.-T., Mosaib M.G., Khalil M.I., Islam M.A., Gan S.H. (2020). Potentials and Safety of Date Palm Fruit against Diabetes: A Critical Review. Foods.

[18] Waly M. (2020). Health Benefits and Nutritional Aspects of Date Palm Pollen. Can. J. Clin. Nutr..

[19] Farag M.A., Otify A., Baky M.H. (2023). *Phoenix Dactylifera* L. Date Fruit By-products Outgoing and Potential Novel Trends of Phytochemical, Nutritive and Medicinal Merits. Food Rev. Int..

[20] Zain M.R.A.M., Kari Z.A., Dawood M.A.O., Ariff N.S.N.A., Salmuna Z.N., Ismail N., Ibrahim A.H., Krishnan K.T., Mat N.F.C., Edinur H.A. (2022). Bioactivity and Pharmacological Potential of Date Palm (*Phoenix dactylifera* L.) Against Pandemic COVID-19: A Comprehensive Review. Appl. Biochem. Biotechnol..

[21] Ahmed H., Inam ur Raheem M., Khalid W., Zubair Khalid M., Sajid Saleem F., Sabir A., Sharif I., Zafar K.-W. (2022). Health Benefits, Male Fertility, Nutritional Aspects of Dates and Date Palm Pollens: An Overview. JPAA.

[22] Shahriarinour M., Divsar F. (2023). Release Kinetics and Antibacterial Property of Curcumin-Loaded Date Palm (*Phoenix dactylifera* L.) Pollen. Arab. J. Sci. Eng..

[23] Elblehi S.S., El-Sayed Y.S., Soliman M.M., Shukry M. (2021). Date Palm Pollen Extract Avert Doxorubicin-Induced Cardiomyopathy Fibrosis and Associated Oxidative/Nitrosative Stress, Inflammatory Cascade, and Apoptosis-Targeting Bax/Bcl-2 and Caspase-3 Signaling Pathways. Animals.

[24] Daoud A., Malika D., Bakari S., Hfaiedh N., Mnafgui K., Kadri A., Gharsallah N. (2019). Assessment of polyphenol composition, antioxidant and antimicrobial properties of various extracts of Date Palm Pollen (DPP) from two Tunisian cultivars. Arab. J. Chem..

[25] Hachef A., Bourguiba H., Cherif E., Ivorra S., Terral J.-F., Zehdi-Azouzi S. (2023). Agro-morphological traits assessment of Tunisian male date palms (Phœnix dactylifera L.) for preservation and sustainable utilization of local germplasm. Saudi J. Biol. Sci..

[26] Kostić A.Ž., Milinčić D.D., Barać M.B., Shariati M., Tešić Ž.L.j., Pešić M.B. (2020). The Application of Pollen as a Functional Food and Feed Ingredient—The Present and Perspectives. Biomolecules.

[27] Al-Mssallem M.Q., Alqurashi R.M., Al-Khayri J.M. (2019). Bioactive Compounds of Date Palm (*Phoenix dactylifera* L.). Bioactive Compounds in Underutilized Fruits and Nuts.

[28] Sporchia F., Patrizi N., Pulselli F.M. (2023). Date Fruit Production and Consumption: A Perspective on Global Trends and Drivers from a Multidimensional Footprint Assessment. Sustainability.

[29] Paszke M.Z. (2019). Date Palm and Date Palm Inflorescences in the Late Uruk Period (*c.* 3300 b.c.): Botany and Archaic Script. Iraq.

[30] Platat C., Habib H.M., Hashim I.B., Kamal H., AlMaqbali F., Souka U., Ibrahim W.H. (2015). Production of functional pita bread using date seed powder. J. Food Sci. Technol..

[31] Habib H.M., Ibrahim W.H. (2011). Effect of date seeds on oxidative damage and antioxidant status *in vivo*. J. Sci. Food Agric..

[32] Habib H.M., El-Fakharany E.M., Souka U.D., Elsebaee F.M., El-Ziney M.G., Ibrahim W.H. (2022). Polyphenol-Rich Date Palm Fruit Seed (*Phoenix Dactylifera* L.) Extract Inhibits Labile Iron, Enzyme, and Cancer Cell Activities, and DNA and Protein Damage. Nutrients.

[33] Al Meqbaali F.T., Habib H., Othman A., Al-Marzooqi S., Al-Bawardi A., Pathan J.Y., Hilary S., Souka U., Al-Hammadi S., Ibrahim W. (2017). The antioxidant activity of date seed: Preliminary results of a preclinical in vivo study. Emir. J. Food Agric..

[34] Habib H.M., Theuri S.W., Kheadr E., Mohamed F.E. (2016). DNA and BSA damage inhibitory activities, and anti-acetylcholinesterase, anti-porcine α-amylase and antioxidant properties of Dolichos lablab beans. Food Funct..

[35] Habib H.M., Ibrahim W.H., Schneider-Stock R., Hassan H.M. (2013). Camel milk lactoferrin reduces the proliferation of colorectal cancer cells and exerts antioxidant and DNA damage inhibitory activities. Food Chem..

[36] Platat C., Habib H., Al Maqbali F.D., Jaber N.N., Ibrahim W.H. (2014). Identification of Date Seeds Varieties Patterns to Optimize Nutritional Benefits of Date Seeds. J. Nutr. Food Sci..

[37] Habib H.M., Al Meqbali F.T., Kamal H., Souka U.D., Ibrahim W. (2014). Bioactive components, antioxidant and DNA damage inhibitory activities of honeys from arid regions. Food Chem..

[38] Habib H.M., El-Fakharany E.M., Kheadr E., Ibrahim W.H. (2022). Grape seed proanthocyanidin extract inhibits DNA and protein damage and labile iron, enzyme, and cancer cell activities. Sci. Rep..

[39] Habib H.M., Theuri S.W., Kheadr E.E., Mohamed F.E. (2016). Functional, bioactive, biochemical, and physicochemical properties of the Dolichos lablab bean. Food Funct..

[40] Bubonja-Šonje M., Knežević S., Abram M. (2020). Challenges to antimicrobial susceptibility testing of plant-derived polyphenolic compounds. Arch. Ind. Hyg. Toxicol..

[41] Dawoud N.T.A., El-Fakharany E.M., Abdallah A.E., El-Gendi H., Lotfy D.R. (2022). Synthesis, and docking studies of novel heterocycles incorporating the indazolylthiazole moiety as antimicrobial and anticancer agents. Sci. Rep..

[42] Saleh A.K., El-Gendi H., El-Fakharany E.M., Owda M.E., Awad M.A., Kamoun E.A. (2022). Exploitation of cantaloupe peels for bacterial cellulose production and functionalization with green synthesized Copper oxide nanoparticles for diverse biological applications. Sci. Rep..

[43] Omer A.M., Eltaweil A.S., El-Fakharany E.M., El-Monaem E.M.A., Ismail M.M.F., Mohy-Eldin M.S., Ayoup M.S. (2023). Novel Cytocompatible Chitosan Schiff Base Derivative as a Potent Antibacterial, Antidiabetic, and Anticancer Agent. Arab. J. Sci. Eng..

[44] El-Fakharany E.M., Abu-Serie M.M., Litus E.A., Permyakov S.E., Permyakov E.A., Uversky V.N., Redwan E.M. (2018). The Use of Human, Bovine, and Camel Milk Albumins in Anticancer Complexes with Oleic Acid. Protein J..

[45] Abu-Serie M.M., El-Fakharany E.M. (2017). Efficiency of novel nanocombinations of bovine milk proteins (lactoperoxidase and lactoferrin) for combating different human cancer cell lines. Sci. Rep..

[46] El-Fakharany E.M., Abu-Serie M.M., Habashy N.H., Eltarahony M. (2022). Augmenting apoptosis-mediated anticancer activity of lactoperoxidase and lactoferrin by nanocombination with copper and iron hybrid nanometals. Sci. Rep..

[47] Hilary S., Habib H., Souka U., Ibrahim W., Platat C. (2017). Bioactivity of arid region honey: An in vitro study. BMC Complement. Altern. Med..

[48] Bouhlali E.D.T., Alem C., Ennassir J., Benlyas M., Mbark A.N., Zegzouti Y.F. (2017). Phytochemical compositions and antioxidant capacity of three date (*Phoenix dactylifera* L.) seeds varieties grown in the South East Morocco. J. Saudi Soc. Agric. Sci..

[49] Bouhlali E.D.T., Derouich M., Meziani R., Bourkhis B., Filali-Zegzouti Y., Alem C. (2020). Nutritional, mineral and organic acid composition of syrups produced from six Moroccan date fruit (*Phoenix dactylifera* L.) varieties. J. Food Compos. Anal..

[50] Bouhlali E.D.T., Hmidani A., Bourkhis B., Khouya T., Ramchoun M., Filali-Zegzouti Y., Alem C. (2020). Phenolic profile and anti-inflammatory activity of four Moroccan date (*Phoenix dactylifera* L.) seed varieties. Heliyon.

[51] Sebii H., Karra S., Bchir B., Ghribi A.M., Danthine S.M., Blecker C., Attia H., Besbes S. (2019). Physico-Chemical, Surface and Thermal Properties of Date Palm Pollen as a Novel Nutritive Ingredient. Adv. Food Technol. Nutr. Sci. Open J..

[52] Zeid H.M.A., Shiha M.A., Shehata A.A. (2019). Comparative Study of Pollen Grains Morphology and Phytochemical Constituents of Some Saudi Arabian Date Palm (*Phoenix dactylifera* L.) Cultivars. Int. J. Curr. Microbiol. Appl. Sci..

[53] Aybastıer Ö., Dawbaa S., Demir C. (2018). Investigation of antioxidant ability of grape seeds extract to prevent oxidatively induced DNA damage by gas chromatography-tandem mass spectrometry. J. Chromatogr. B.

[54] El-Kholy W.M., Soliman T.N., Darwish A.M.G. (2019). Evaluation of date palm pollen (*Phoenix dactylifera* L.) encapsulation, impact on the nutritional and functional properties of fortified yoghurt. PLoS ONE.

[55] Echegaray N., Pateiro M., Gullón B., Amarowicz R., Misihairabgwi J.M., Lorenzo J.M. (2020). Phoenix dactylifera products in human health—A review. Trends Food Sci. Technol..

[56] Kruk J., Aboul-Enein B.H., Duchnik E., Marchlewicz M. (2022). Antioxidative properties of phenolic compounds and their effect on oxidative stress induced by severe physical exercise. J. Physiol. Sci..

[57] Hossain M.B., Ahmed L., Martin-Diana A.B., Brunton N.P., Barry-Ryan C. (2023). Individual and Combined Antioxidant Activity of Spices and Spice Phenolics. Antioxidants.

[58] Chelliah R., Banan-MwineDaliri E., Oh D.-H. (2022). Screening for Antioxidant Activity: Total Antioxidant Assay. Methods in Actinobacteriology.

[59] Apak R., Calokerinos A., Gorinstein S., Segundo M.A., Hibbert D.B., Gülçin I., Çekiç S.D., Güçlü K., Özyürek M., Çelik S.E. (2021). Methods to evaluate the scavenging activity of antioxidants toward reactive oxygen and nitrogen species (IUPAC Technical Report). Pure Appl. Chem..

[60] McDonald A.G., Tipton K.F. (2020). Enzymes: Irreversible Inhibition. Encyclopedia of Life Sciences.

[61] Habib H.M., Kheadr E., Ibrahim W.H. (2021). Inhibitory effects of honey from arid land on some enzymes and protein damage. Food Chem..

[62] Yu Q., Fan L. (2021). Understanding the combined effect and inhibition mechanism of 4-hydroxycinnamic acid and ferulic acid as tyrosinase inhibitors. Food Chem..

[63] Zheng Y., Yang W., Sun W., Chen S., Liu D., Kong X., Tian J., Ye X. (2019). Inhibition of porcine pancreatic α-amylase activity by chlorogenic acid. J. Funct. Foods.

[64] Niu F., Xie W., Zhang W., Kawuki J., Yu X. (2023). Vitamin C, vitamin E, β-carotene and risk of Parkinson’s disease: A systematic review and dose–response meta-analysis of observational studies. Nutr. Neurosci..

[65] Miethke M., Pieroni M., Weber T., Brönstrup M., Hammann P., Halby L., Arimondo P.B., Glaser P., Aigle B., Bode H.B. (2021). Towards the sustainable discovery and development of new antibiotics. Nat. Rev. Chem..

